# An Asynchronous, Mobile Text-Based Platform (XatJove Anoia) for Providing Health Services to Teenagers: Protocol for a Quasiexperimental Study

**DOI:** 10.2196/25062

**Published:** 2021-02-03

**Authors:** Glòria Sauch Valmaña, Josep Vidal-Alaball, Victoria Garcia Furió, Giorgia Testoni, Albert Espelt, Katarin Exposito, Francesc Saigí-Rubió, Núria Carré, Ikuska Sanz, Victor Vicens

**Affiliations:** 1 Health Promotion in Rural Areas Research Group Gerència Territorial de la Catalunya Central Institut Català de la Salut Sant Fruitós de Bages Spain; 2 Unitat de Suport a la Recerca de la Catalunya Central Fundació Institut Universitari per a la recerca a l'Atenció Primària de Salut Jordi Gol i Gurina Sant Fruitós de Bages Spain; 3 Primary Care Center Igualada Gerència Territorial de la Catalunya Central Institut Català de la Salut Igualada Spain; 4 Abi Global Health Limited Dublin Ireland; 5 Faculty of Health Science Universitat de Vic Universitat Central de Catalunya Manresa Spain; 6 Centro de Investigación Biomédica en Red Epidemiologia y Salud Pública Madrid Spain; 7 Santa Coloma de Queralt Primary Care Center Gerència Territorial de la Catalunya Central Institut Català de la Salut Santa Coloma de Queralt Spain; 8 Faculty of Health Sciences Universitat Oberta de Catalunya Barcelona Spain; 9 Primary Care Regional Service Anoia Gerència Territorial de la Catalunya Central Institut Català de la Salut Igualada Spain

**Keywords:** mHealth, telehealth, teenager, health promotion and sexual health, health promotion, sexual health

## Abstract

**Background:**

Due to the COVID-19 pandemic, it is more essential than ever to implement protective measures in primary care centers to ensure patients’ safety. This protocol describes a quasiexperimental study on the use of a mobile chat platform as a clinical consultation tool for adolescents and primary health care physicians.

**Objective:**

The purpose of the quasiexperimental study is to demonstrate that the use of mobile phones and messaging apps increases the number of health consultations. The study will be performed as part of the Health and School program in the Anoia region.

**Methods:**

The quasiexperimental study will compare the number of face-to-face consultations to the number of consultations conducted on XatJove Anoia, as part of the Health in Schools program in the Anoia region. The study will involve the use of a new communication platform (ie, XatJove Anoia) for health care professionals and adolescents, and data on the number of face-to-face consultations will be collected as part of the same program in another region. Data will be collected from secondary schools during the academic year 2020-2021. Statistical analyses will be performed on the data that users will enter in the registration form. These data will be collected by means of a questionnaire, which will be submitted once the questionnaire is closed. The questionnaire will consist of multiple-choice questions, which will allow numerical values to be assigned to various responses in order to carry out statistical analyses.

**Results:**

The study is projected to start at the beginning of November 2020 and finish in June 2021, which is when data analysis is expected to start.

**Conclusions:**

The results of the quasiexperimental study may assist in the development and planning of school health programs.

**Trial Registration:**

ClinicalTrials.gov NCT04562350; https://clinicaltrials.gov/ct2/show/NCT04562350.

**International Registered Report Identifier (IRRID):**

PRR1-10.2196/25062

## Introduction

The World Health Organization defines “digital health” as the use of digital technologies for health purposes; digital health is a category that encompasses the increasing use of technologies for health services [1]. The internet has become an important tool for many people with health concerns, especially adolescents. Concerns regarding confidentiality, coupled with the stigma and shame associated with certain conditions, such as sexually transmitted infections and other health-related problems, make the internet an unsafe environment for adolescents who seek information.

Sex education is essential for the prevention of risky sexual behaviors, unwanted pregnancies, and the transmission of human immunodeficiency viruses and other sexually transmitted infections among adolescents. Young people receive sexual and reproductive health education from various sources, including formal education, school curricula, parents, fellow students, and media [2].

During the International Conference on Population and Development [2], several measures were established as a response to the need for providing relevant information that protects the sexual and reproductive lives of young people. Governments have been requested to provide sex education policies to promote the well-being of young people at a community level, especially in the school environment. Such policies will allow young people to make mature decisions regarding responsible sexual behavior much earlier than usual [3].

Finding information on health-related issues can be a major problem, as the underutilization of primary care services that provide information on certain topics and the untrustworthy nature of advice on the internet can subsequently lead to health-related complications that may require expensive and specialized medical interventions in the long term. These complications can ultimately lead to an increase in health care costs [4].

The Salut i Escola program (ie, the Health in Schools program) was launched by the Catalan Department of Health in the academic year 2004-2005. This program aims to improve the health of adolescents in Catalonia. The program involves health promotion, risk prevention, and early detection for problems related to mental health, emotional and sexual health, and drug, alcohol, and tobacco consumption. The program has been carried out in close collaboration with local schools and community health services.

This community-based outreach program requires intervention and cooperation from different professionals. One of the program’s main lines of activity is the open consultation service, in which a health care professional visits schools to facilitate primary care accessibility among adolescents and guarantee privacy, confidentiality, and proximity.

Patients—particularly adolescent patients—are often reluctant to seek counselling and health treatment for embarrassing or stigmatized conditions, which can result in the underutilization of primary care health services. In addition, searching for medical information on the internet (eg, searching via Google) is increasingly common. Primary care health services, which are often made for young people, include consultations for sexual and reproductive health problems [5] and mental health disorders [6]. Finding information on health-related issues can lead to serious problems; the underutilization of primary care services that provide information on certain issues and the untrustworthy nature of advice on the internet can subsequently lead to health complications that may require expensive and specialized medical interventions in the long run, which may result in increased care costs [4].

To avoid health complications and increased care costs, a tool that adolescents have easy, constant, and effective access to is required. Digital health interventions have been shown to minimize hesitancy in seeking health advice for stigmatized and embarrassing problems. The digital health innovations proposed by the company Abi Global Health (AGH) could provide a possible solution [7]. AGH has developed an asynchronous, mobile text-based communication platform that connects users to health care professionals. These professionals provide users with appropriate guidance to help them make informed decisions about their health. AGH operates in more than 10 countries and has a network of more than 300 health professionals. This preexisting tool will form the basis of XatJove Anoia (ie, YouthChat Anoia), which is a platform that attempts to provide resources for mobile communication and message exchanges between adolescents and primary care professionals. A quasiexperimental study on XatJove Anoia will take place in Anoia, which is a country in Central Catalonia.

According to a pilot study, cystitis and contraceptive problems are among the top 10 reasons why patients have used a web-based tool to conduct a consultation [8]. Although these conditions can be embarrassing and difficult to talk about, it is important that they are dealt with by health care professionals. If ignored, these conditions can lead to serious, unwanted, and costly complications (eg, a failure to use contraception can lead to unwanted pregnancies and sexually transmitted diseases) [9,10]. The fact that users have routinely reported these problems by means of digital health interventions, such as e-consulta (ie, an asynchronous teleconsulting service for primary care professionals and health service users that is connected to primary care electronic medical records), is a positive sign with regard to the greater use of health services for sensitive and serious conditions. As an anonymous digital health intervention, XatJove has the potential to address the inefficient costs resulting from the underutilization of health services for embarrassing and stigmatized disorders.

Due to the current climate of the health crisis caused by the COVID-19 pandemic [11] and the need to reduce the risk of infection, it makes more sense than ever to avoid face-to-face consultations with nursing staff in schools and primary care center visits by young people. It should be noted that this does not mean that there will be a reduction in the number of consultations for health-related issues and the number of school programs. On the contrary, the health crisis is expected to generate an increase in the need for emotional support for young people, and such support requires tools that allow for the quick use of technology (ie, technology that adolescents and primary care center professionals are familiar with and use regularly).

The main objective of this protocol is to describe a quasiexperimental study that shows that the use of mobile phones and messaging apps leads to an increase in the number of health consultations for adolescents aged between 12 and 16 years, as part of the Health and School program in the Anoia region. The study also seeks to evaluate the degree of satisfaction among XatJove users via an electronic survey.

Our main hypothesis is that the use of XatJove will improve the early detection of health problems, the accessibility of reliable information for young people, and communication among nursing professionals in primary care centers, in terms of issues related to drugs, diet, emotional health, and sexuality. We also believe that XatJove can help with the detection of child abuse cases.

## Methods

### Study Design

A quasiexperimental study that compares the total number of face-to-face consultations and XatJove consultations will be conducted as part of the Health and School program in the Anoia region (ie, the intervention group) during the 2020-2021 academic year. Data on the number of face-to-face consultations will be collected from the same program in the Osona region (ie, the control group). Problems that affect adolescents and relate to mental health, emotional and sexual health, drug use, alcohol, and tobacco will be recorded. The study population will include adolescents aged between 12 and 16 years who attend secondary schools in the Anoia region. We will use a pragmatic sample of 100 XatJove consultations. The total number of visits (ie, face-to-face visits and XatJove visits) that relate to the Health and School program in the Anoia region during the 2020-2021 academic year will be recorded.

The following variables will be recorded: (1) universal variables, including gender and age; (2) dependent variables, including the total number of visits (ie, face-to-face visits and XatJove visits) that relate to the Health and School program; and (3) independent variables, including the topic of consultations (ie, consultations for sexual health, alcohol, drugs, eating disorders, bullying, domestic abuse, mental health, COVID-19, and other topics).

To learn more about the usefulness of XatJove, students will be asked to participate in a follow-up study that involves a focus group.

### Data Collection and Sources of Information

Data regarding the total number of visits will be obtained from the Primary Care Services Information Technologies System (ie, Sistemes d’Informació dels Serveis d’Atenció Primària in Catalan), which belongs to the Catalan Institute of Health in Barcelona, Spain.

The asynchronous, mobile text-based communication platform that is made available by AGH makes it possible to collect information on cases that involve a written query. User data will be obtained from the platform’s database, and users will remain anonymous. The information collected will include age, gender, the date and time of the query, and the answer to the query. In addition, all the professionals involved in the study will sign a document, in which they will agree to respect data confidentiality.

After a response from a health care professional is received, users will be able to respond to a questionnaire, in which they can evaluate the quality of the service and their level of satisfaction (Multimedia Appendix 1). The data in this questionnaire will be collected and processed by AGH.

### Statistical Analysis

Spreadsheets will be used to record the means and medians of various data. Statistical analyses will be performed on the data that users will enter in the registration form and the data collected from the questionnaire. The questionnaire will be sent to participants after their consultation concludes. The questionnaire consists of multiple-choice questions, which makes it possible to assign a numerical value to various responses. These values will be used in the corresponding statistical analyses.

### Limitations of the Study

It is possible that an insufficient number of users will rate the service and respond to the questionnaire. If this happens, the deadline for collecting data will be extended.

Although it will be possible to send questions via XatJove 24 hours per day, answers will only be sent during specific time slots (ie, from 8 AM to 8 PM, including weekends). This may limit the accessibility of the service. However, any inquiries made outside of this time slot will be answered by medical professionals at the start of their next working day.

Since XatJove is an anonymized service, it will not be possible to verify whether users meet the inclusion criteria. Nevertheless, the service will only be offered to students who attend secondary schools in Anoia.

### Ethical Considerations

The Institut Universitari d’Investigació en Atenció Primària Jordi Gol independent ethics committee in Barcelona, Spain approved the trial study protocol (code 20/137-P). Written informed consent will be requested from all patients who participate in the study. The study was registered on the ClinicalTrial.gov registry (NCT04562350) on September 24, 2020.

### Availability of Data and Materials

All principal investigators of the study will have access to the complete dataset. The datasets generated and analyzed during the study will be made available by the corresponding author. The results generated during the study will be published in peer-reviewed journals and presented at national/international congresses.

The study has been designed in accordance with the CONSORT (Consolidated Standards of Reporting Trials) guidelines. The registration of the study on ClinicalTrials.gov is expected to facilitate transparency and reporting.

## Results

The study is projected to start at the beginning of November 2020 and finish in June 2021, which is when data analysis is expected to start.

## Discussion

This aim of the pilot quasiexperimental study is to investigate the use of a mobile phone health chat service for adolescents from secondary schools in Anoia and nursing professionals from primary care centers in the same region.

The various studies that we previously participated in have shown that the use of a web-based communication tool for patients and professionals reduces the number of face-to-face visits [12,13], which is a very positive aspect with regard to the COVID-19 pandemic. Furthermore, the results of the study and the long-term and short-term impacts of XatJove will be used to revise the Health and School program, which is offered by the Department of Health. In other words, XatJove is a tool that will serve in the review of existing protocols. Web-based visits and consultations are gaining in popularity in the new health care environment that has resulted from the COVID-19 pandemic.

Web-based consultations can help with avoiding self-diagnoses, as it is common for people to search for information on the internet, which is not always a reliable source. Current evidence points to the importance of innovating and improving the treatment processes offered by health and school programs. One of our study’s strengths is that the results will be obtained from usual clinical practices, without having to implement any considerable organizational or structural changes.

## Appendix

Multimedia Appendix 1 Questionnaire for evaluating the quality of the service.

## Abbreviations

AGH: Abi Global Health
CONSORT: Consolidated Standards of Reporting Trials
