# Supplementary material for: An Asynchronous, Mobile Text-Based Platform (XatJove Anoia) for Providing Health Services to Teenagers: Protocol for a Quasiexperimental Study
Source: JMIR Res Protoc. 2021 Feb 3;10(2):e25062. doi: 10.2196/25062 (PMC7889420; doi:10.2196/25062)
Supplement: Multimedia Appendix 1 [file resprot_v10i2e25062_app1.docx]

1- Age in years:

2- Sex: M, F, other

3- Have we helped you solve a problem related to your health or well-being?

(1 = insufficient; 5 = excellent)

4- Would you have made an appointment with your PCC or the Health and School program for this problem? (yes/no)

5- Would you recommend this service to a friend, family member or acquaintance? (1 = no; 5 = strongly agree)

6- Would you be prepared to take part in a follow up study on *XatJove*? If so, please provide an email address or mobile phone number so we can contact you.
